# Hepatitis B infection in the general population of China: a systematic review and meta-analysis

**DOI:** 10.1186/s12879-019-4428-y

**Published:** 2019-09-18

**Authors:** Huai Wang, Peixuan Men, Yufeng Xiao, Pei Gao, Min Lv, Qianli Yuan, Weixin Chen, Shuang Bai, Jiang Wu

**Affiliations:** 1 Institute for immunization and prevention, Beijing Center for Disease Prevention and Control, Beijing Research Center for Preventive Medicine, No.16, HePingLi Middle Street, DongCheng District, Beijing, 100013 China; 2 China Institute of Medical Information/Medical Library, CAMS&PUMC, No. 69 Dongdan North Street, Dongcheng District, Beijing, 100005 China

**Keywords:** Hepatitis B, Meta-analysis, Prevalence, China

## Abstract

**Background:**

Hepatitis B virus (HBV) infection is a major public health problem in China. Over a decade has passed since the last National Hepatitis Seroepidemiological Survey was conducted in 2006. The lack of updated data on hepatitis B in China makes assessing the current prevalence and burden of the disease inadequate. In response to the above situation, a systematic review and meta-analysis was conducted to provide a better understanding of hepatitis B epidemiology in the general population of China.

**Methods:**

A systematic search was conducted in international databases (Medline through PubMed, EMBASE, Cochrane, Web of Science) and national databases (CBM, CNKI, WanFang Data) to retrieve primary studies published between January 1, 2013 and December 31, 2017. The pooled prevalence of HBV infection and 95% confidence intervals were calculated. Quality assessment, heterogeneity testing and publication bias assessment were also performed.

**Results:**

Of the 27 studies included in the meta-analysis, the pooled estimated prevalence of HBV infection in the general population of China from 2013 to 2017 was 6.89% (95% *CI*:5.84–7.95%), which could be extrapolated to an estimated population of 84 million living with HBsAg in 2018. The prevalence of HBV infection in males was higher than that in females (5.88% vs 5.05%), and rural areas had a higher prevalence than urban areas (5.86% vs 3.29%). The highest prevalence of HBV infection was reported in Western provinces (8.92, 95% CI: 7.19–10.64%). In adults older than 20 years, the prevalence of HBV infection was approximately 7%, which was higher than that in children.

**Conclusion:**

The prevalence of HBV infection in the general population of China was classified as higher intermediate prevalence (5–7.99%), of which more than 90% of the HBV infection population included adults older than 20 years. The blocking of mother-to-infant hepatitis B transmission and plans involving timely birth dose of hepatitis B vaccine within 24 h should be implemented. Additionally, improving the quality of life and survival rate of the infected population through antiviral therapy and high-risk adult vaccination will be the priority of our future work. Moreover, various control measures should be implemented in different provinces across China.

**Electronic supplementary material:**

The online version of this article (10.1186/s12879-019-4428-y) contains supplementary material, which is available to authorized users.

## Background

Hepatitis B virus (HBV) infection is a major global public health problem and nearly 2.57 billion people worldwide are estimated to be infected with HBV [1, 2]. People chronically infected with HBV are at an increased risk of developing HBV-related liver diseases, including hepatic cirrhosis and hepatocellular carcinoma (HCC) [3, 4]. Without a more extensive prevention method, it is expected that the number of people infected with hepatitis B will remain at the current level. The estimated annual mortality of hepatitis B is more than 780,000 worldwide [1, 2]. The frequency of HBV infection is different throughout the world [5]. High prevalence areas are defined as areas wherein more than 8% of the population is positive for the hepatitis B surface antigen (HBsAg); higher intermediate prevalence areas are defined as areas wherein 5–7.99% of the population is HBsAg positive; lower intermediate prevalence areas are defined as areas wherein 2–4.99% of the population is HBsAg positive; and low prevalence areas are defined as areas wherein less than 2% of the population is HBsAg positive [5–7].

In 1992, the first Chinese National Hepatitis Seroepidemiological Survey found that the prevalence of HBsAg among population aged 1–59 years was 9.75%, which indicated a hepatitis B high prevalence area. Based on this survey, it was estimated that 120 million people carried HBsAg, 20 million suffered from chronic hepatitis B, and almost 300,000 died annually from chronic consequences of HBV infection in China [8–10]. Since then, the Ministry of Health has recommended hepatitis B vaccine for routine immunization of infants in 1992 and has integrated hepatitis B vaccine into EPI in 2002, with an emphasis on providing a timely birth dose (within 24 h of birth) [11]. In 2006, the second Chinese National Hepatitis Seroepidemiological Survey found that the prevalence of HBsAg for population aged 1–59 years decreased to 7.18%, which indicated that an estimated 16–20 million HBV carriers were prevented from infection through hepatitis B vaccination of infants [11]. However, it was estimated that there were more than 93 million chronic HBV infections in 2006 which resulted in a public health issue [11]. From 2006 to 2017, over a decade has passed. Unfortunately, no investigations have been conducted at the country level. Aparna Schweitzer systematically reviewed the status of chronic HBV infection around the world from 1965 to 2013 and estimated that the prevalence of hepatitis B infection in China was 5.49% until 2013 [5]. The lack of updated data on hepatitis B in China makes assessing the current prevalence and burden of the disease inadequate, and results in ineffective policy making. Therefore, a systematic review and meta-analysis based on data published in the last 5 years (2013–2017) was conducted to provide a better understanding of hepatitis B epidemiology in the general population of China.

## Methods

### Search strategy

This systematic review and meta-analysis on hepatitis B prevalence data was conducted following the criteria of the PRISMA (Preferred Reporting Items for Systematic Reviews and Meta-Analyses) statement guidelines [12]. International databases (Medline through PubMed, EMBASE, Cochrane, and Web of Science) and national databases (Chinese Biomedical Database (CBM), China National Knowledge Infrastructure (CNKI), and WanFang Data) were searched for all published literature between January 1, 2013 and December 31, 2017. During the search, the following keywords were used: [“HBsAg” OR “hepatitis B” OR “Hepatitis B virus” OR “HBV”] AND [“prevalence” OR “prevalent” OR “epidemic” OR “epidemiology” OR “carrier” OR “positive rate” OR “infection rate”] AND [“Chinese” OR “China”] AND [(“2013”[Date - Publication]: “2017”[Date - Publication])].

### Inclusion and exclusion criteria

All articles that reported cross-sectional studies on the general population tested for HBsAg in different regions of China were included. All articles were published from 2013 to 2017. The inclusion criteria were restricted to original research articles published in English or Chinese. The exclusion criteria were the following: (1) conference abstracts, case reports, surveillance reports, and systematic reviews or meta-analyses; (2) study designes with non-random sampling; (3) studies that did not include the positive rate of HBsAg; (4) sample sizes of less than 800 people or studies conducted in only a rural area; (5) study populations coinfected with HBV, HCV and HIV.

### Data extraction and quality assessment

The data were extracted by two independent reviewers (GP and YQL). The titles and abstracts were screened for relevance. After a review of the full-text articles, the following data were extracted from each study: study name, first author, publication year, study design, province or city, sex, age, sample size, number of HBsAg positive individuals and HBsAg test methods. The references of all identified full-text articles were also checked to identify whether there were any additional articles that were missed during screening. Each of the two independent reviewers read the full-text articles and extracted the data. Any inconsistencies and disagreements between the two independent reviewers were resolved through discussion or consultation with the third reviewer (WH).

The quality of all included articles was assessed using the Cross-Sectional/Prevalence Study Quality Assessment Forms which were recommended by the Agency for Healthcare Research and Quality (AHRQ) [13]. In the AHRQ form (Additional file 1: Table S1), there are eleven questions, of which ten questions are fitted for cross-sectional studies, i.e., the questions were answered with “Yes”, “No” and “Unclear”. “Yes” represented a score of 1 and “No” or “Unclear” represented a score of 0. The last question was fitted for follow-up studies was not covered in our study. In the meta-analysis, the total score of the ten questions in the AHRQ form was used to assess the quality of each full-text article.

### Statistical analysis

Statistical analysis was performed by Stata software (Version 13.0, Stata Corp, College Station, TX, USA). The prevalence rate of hepatitis B was defined as the positive rate of HBsAg. Individual proportions and the HBV pooled prevalence were assessed at 95% confidence interval (95% CI) and was showed by a forest plot. We performed subgroup analyses on all articles according to different regions, age groups, sexes, and urban or rural areas. According to the geographical location, China is divided into three parts: Eastern, Middle and Western. According to household registration statistics, China is divided into rural and urban areas. Heterogeneity testing was performed using the degree of inconsistency(*I*^*2*^). The degree of heterogeneity was classified to three levels (minimal, *I*^*2*^ < 25%; moderate, 25% ≤ *I*^*2*^ < 50%; substantial, and *I*^*2*^ ≥ 50%) [14]. If no significant heterogeneity was detected (*P* > 0.05 and *I*^*2*^ < 50%), a fixed effect model was used to calculate the HBV pooled prevalence and 95% CI. Otherwise, a random effect model was used. Subgroup analysis was used to minimize heterogeneity and provided more details on the HBV infection. Publication bias was assessed graphically by funnel plot and formally by Egger’s test (significance at *P* < 0.05) [15]. The national total population data in 2018 were from the National Bureau of Statistics.

## Results

### Study general scope

**Fig. 1:**
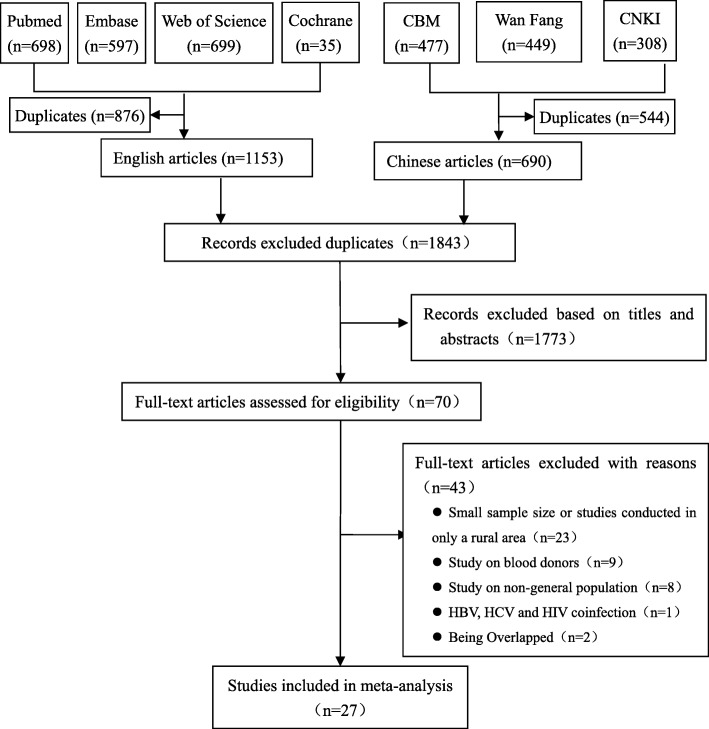
Flow diagram of study selection

A total of 1151 English articles and 690 Chinese articles were identified through 7 database searches. Following the removal of duplicates, primary screening and screening titles or abstracts, 70 publications were reviewed in full text. A total sample size of 5,422,405 people in 27 articles met the eligibility criteria and were therefore included in the meta-analysis (Fig. 1). All included articles [16–42] were cross-sectional studies (Table 1). The sample size was between 904 and 1,966,013. The regional distributions were Eastern (including 16 studies), Middle (including 7 studies) and Western (including 9 studies). The area (urban/rural) distribution was urban (including 10 studies) and rural (including 16 studies). In terms of quality, all studies (100%) had a total quality score higher than 4. 17 studies in 27 studies (62.96%) had a total quality score higher than 5 (Additional file 2: Table S2).

**Table 1:** Characteristics of the studies included in the meta-analysis

Author, Year	Year of Publication	Study Design	Province or City	Age group	Sample Size(N)	HBsAg Positive(n)	Method	Quality Grade
Min DY et al. [16]	2016	Cross-sectional	Gui Zhou province	≥16	1629	136	TRFIA	4
Xia W et al. [17]	2015	Cross-sectional	Hu Nan province	1–59	9536	498	ELISA	4
Cai HL et al. [18]	2017	Cross-sectional	Ning Xia province	55–79	6582	919	ELISA	4
Bai S et al. [19]	2017	Cross-sectional	Tibet province	≥1	904	181	Colloidal Gold	4
Wang FZ et al. [20]	2017	Cross-sectional	31 provinces	1–29	19,583	517	ELISA	5
Chen YH et al. [21]	2017	Cross-sectional	Quan Zhou City	1–59	5473	370	ELISA	5
Gao P et al. [22]	2016	Cross-sectional	Beijing City	≥1	6705	184	CMIA	6
Su FY et al. [23]	2015	Cross-sectional	Jiang Yin City	≥1	13,837	695	ELISA	4
Yang BF et al. [24]	2013	Cross-sectional	Hu Bei province	1–59	9955	676	ELISA	7
Liu J et al. [25]	2014	Cross-sectional	ChongQing City	20–59	21,424	1718	ELISA	5
Ren H et al. [26]	2013	Cross-sectional	Shang Hai City	> 0	2835	172	ELISA	4
Cheng JQ et al. [27]	2013	Cross-sectional	Shen Zhen City	1–59	3771	252	ELISA	4
He HY et al. [28]	2014	Cross-sectional	Tian Jin City	1–59	2594	68	ELISA	5
Guo YH et al. [29]	2017	Cross-sectional	He Nan province	18–74	16,685	642	ELISA	6
Liu J et al. [30]	2017	Cross-sectional	31 provinces	21–49	1,936,801	202,816	ELISA	5
Liu JY et al. [31]	2017	Cross-sectional	Shan Dong province	1–59	5528	187	ELISA	6
Yang SG et al. [32]	2017	Cross-sectional	Zhe Jiang province	0–81	16,601	670	CMIA	6
Chen P et al. [33]	2017	Cross-sectional	Zhe Jiang province	> 0	9855	1056	ELISA	8
Zeng FF et al. [34]	2016	Cross-sectional	Guang Dong province	> 0	169,211	14,823	ELISA	7
Xin XN et al. [35]	2016	Cross-sectional	31 provinces	20–49	764,460	44,057	ELISA	5
Liu J et al. [36]	2016	Cross-sectional	31 provinces	21–49	1,966,013	124,274	ELISA	4
Zhang Q et al. [37]	2016	Cross-sectional	Ji Lin province	≥1	227,808	13,979	ELISA	6
Guo YH et al. [38]	2015	Cross-sectional	He Nan province	1–14	13,207	98	ELISA	5
Huang P et al. [39]	2015	Cross-sectional	Jiang Su province	> 0	148,931	11,469	ELISA	5
Ji ZH et al. [40]	2014	Cross-sectional	Gan Su province	1–59	28,044	2019	ELISA	5
Liao XY et al. [41]	2014	Cross-sectional	Guang Xi province	17–27	2040	258	CMIA	4
Zhang Y et al. [42]	2013	Cross-sectional	Hai Nan province	15–49	12,393	1179	ELISA	4

### Prevalence of HBV infection in the general population

**Fig. 2:**
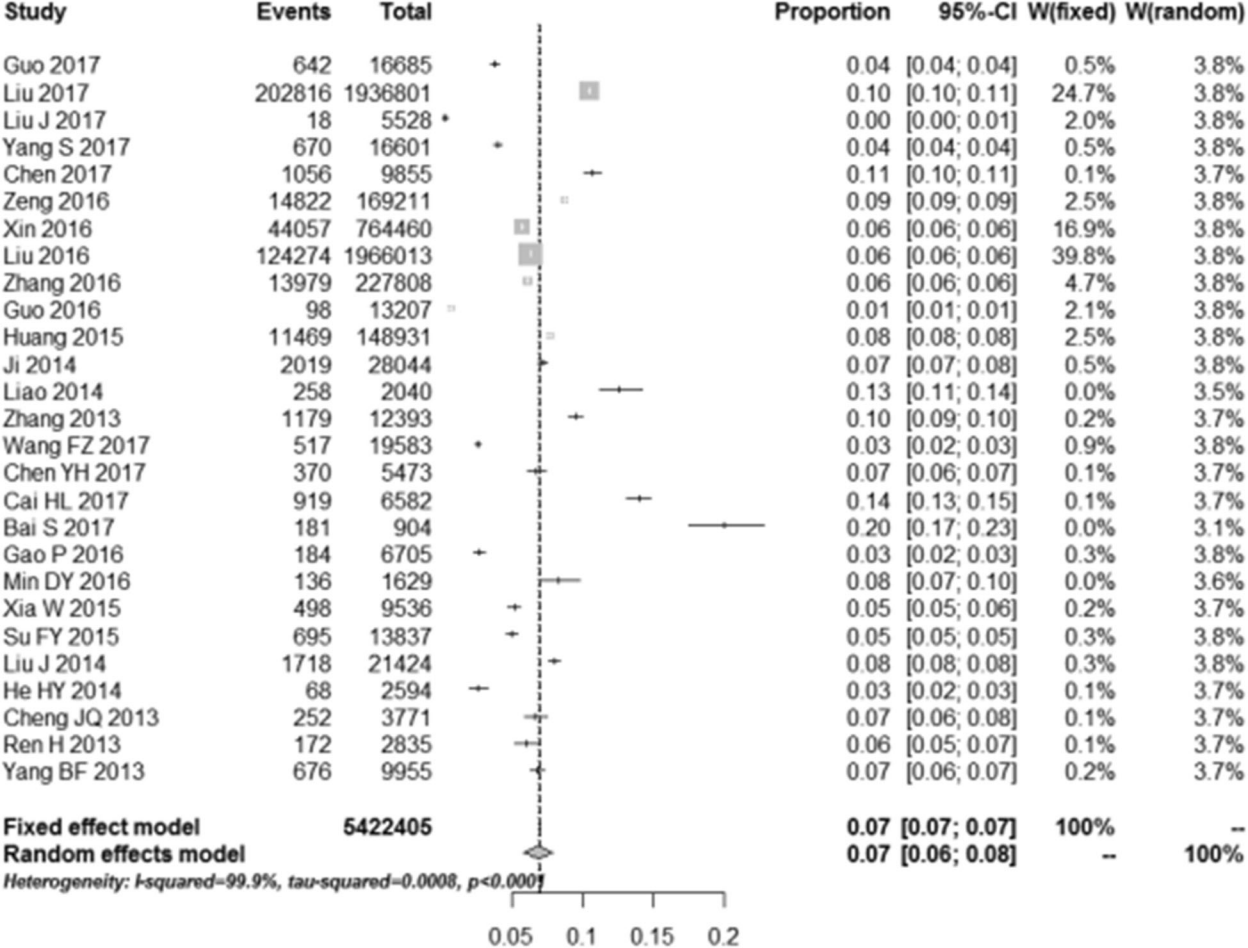
Forest plot of HBV infection prevalence rate in the general Chinese population from 2013 to 2017

In the 27 articles included in our study, the pooled estimated prevalence of HBV infection in the general population of China from 2013 to 2017 was 6.89% (95% *CI*,5.84–7.95%) (Fig. 2). The highest prevalence of HBV infection (20.02%) was reported in Tibet province. The lowest prevalence of HBV infection (2.62%) was reported in Tian Jin city.

**Fig. 3:**
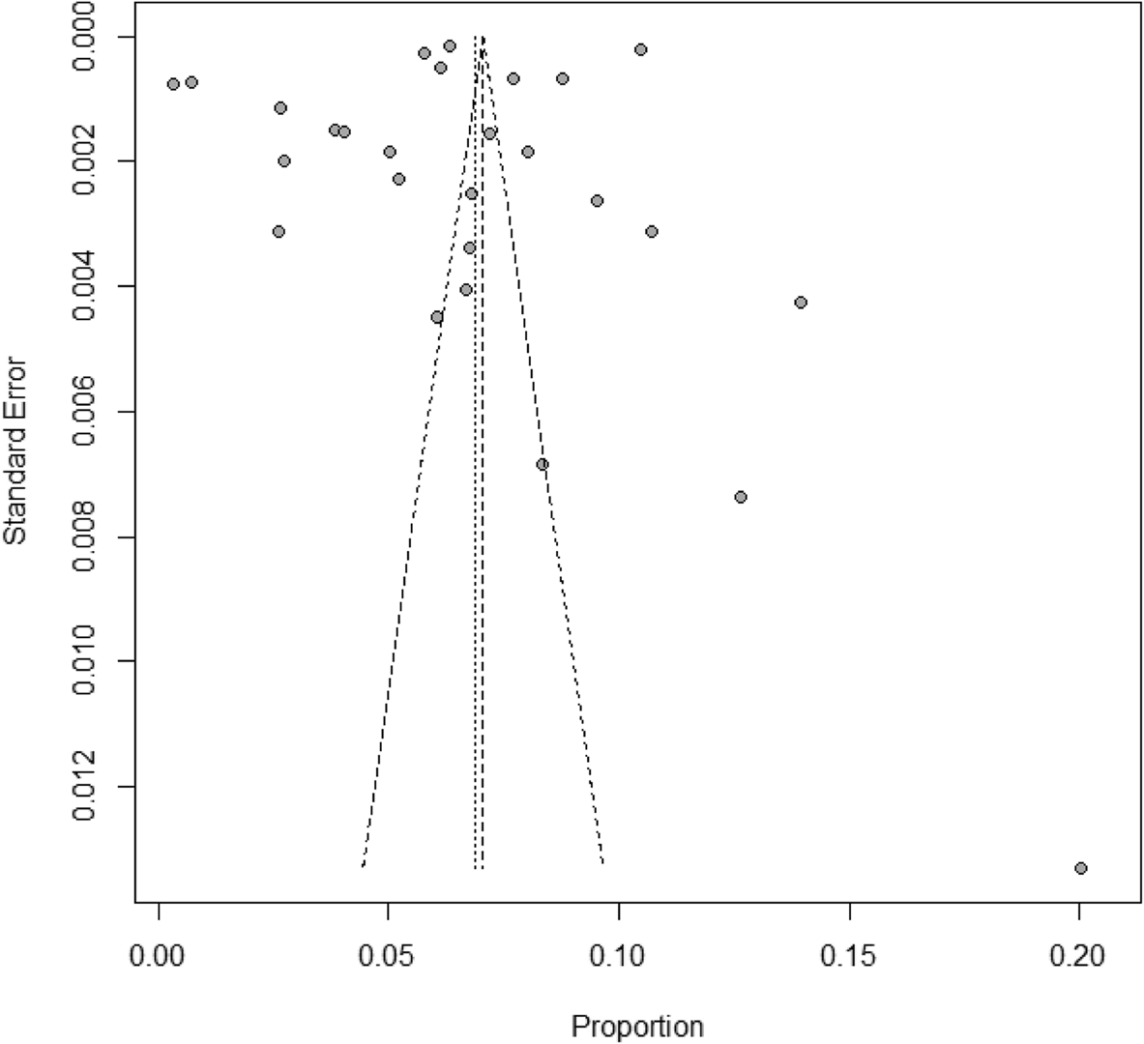
Bias assessment funnel plot of studies reporting HBV prevalence in China from 2013 to 2017

The results of heterogeneity test indicated that the studies were significantly heterogeneous (*I*^*2*^ = 99.9%, *P* < 0.0001). Therefore, a random effect model was used to calculate the HBV pooled prevalence and 95% CI. Based on the funnel plot (Fig. 3) and Egger’s test for the prevalence of HBV infection (*P* = 0.35), there was no evidence of publication bias in all studies.

### Subgroup analysis of HBV infection according to regions, age groups, sexes, and urban or rural areas

The subgroup meta-analysis of pooled HBV infection prevalence is shown in Table 2. The pooled estimated prevalence of HBV infection showed significant differences according to region. The prevalence was the highest in Western China (8.92, 95% CI:7.19–10.64%), which was considered a high prevalence area. A lower prevalence was found in Eastern (6.61, 95% CI:5.27–7.22%) and Middle (5.23, 95% CI:3.11–7.34%) regions, which were considered higher intermediate prevalence areas. The prevalence in males (5.88, 95% CI:5.53–6.24%) was higher than that in females (5.05, 95% CI:4.56–5.88%). The prevalence in children younger than 15 years old was lower than 2%, while in adults older than 20 years old, the prevalence was approximately 7%. The prevalence in rural areas (5.86, 95% CI: 4.93–6.96%) was higher than that in urban areas (3.29, 95% CI: 2.32–4.41%).

**Table 2:** Sub-group meta-analysis of studies reporting HBV prevalence in China from 2013 to 2017

Sub-group	Numbers of Study	Prevalence (%)	95% CI	*I*^*2*^(%)	Heterogeneous *P*-value	Egger’s test P-value*
Region						
Eastern	16	6.16	5.25–7.22	99.9	*P* < 0.0001	0.14
Middle	7	5.23	3.11–7.34	100.0	*P* < 0.0001	–
Western	9	8.92	7.19–10.64	99.9	*P* < 0.0001	–
Sex						
Male	21	5.88	5.53–6.24	99.1	*P* < 0.0001	0.90
Female	22	5.05	4.56–5.88	99.6	*P* < 0.0001	0.51
Age						
0–4	8	0.51	0.33–0.74	50.6	0.048	–
5–9	6	2.08	1.05–3.46	90.2	*P* < 0.0001	–
10–14	6	1.58	0.69–2.77	89.8	*P* < 0.0001	–
15–19	6	3.98	1.42–6.54	96.9	*P* < 0.0001	–
20–29	12	7.07	5.62–8.67	99.9	*P* < 0.0001	0.77
30–39	12	7.43	6.11–9.04	99.9	*P* < 0.0001	0.72
40–49	11	7.08	5.72–8.77	99.2	*P* < 0.0001	0.91
50–59	6	7.08	5.19–9.65	93.2	*P* < 0.0001	–
≥ 60	3	7.39	4.20–10.59	96.7	*P* < 0.0001	–
Urban/Rural						
Urban	10	3.29	2.32–4.41	98.2	*P* < 0.0001	0.52
Rural	16	5.86	4.93–6.96	100.0	*P* < 0.0001	0.45

*: When the sample size is less than 10 articles, it is not possible to do Egger’s test

### Estimation of the number of people living with HBsAg in China

**Fig. 4:**
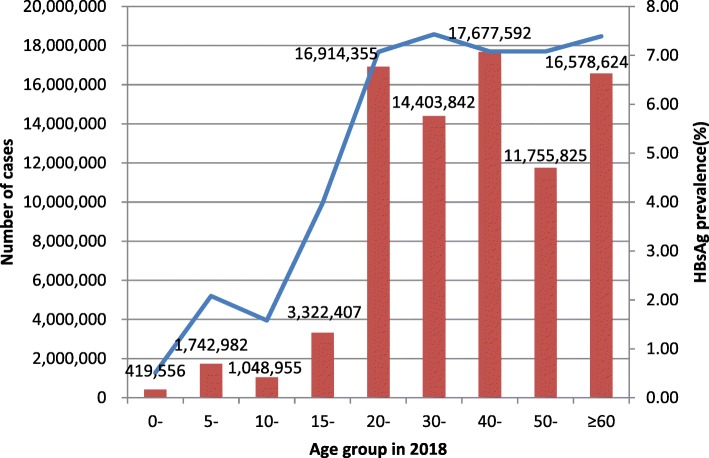
Estimated HBsAg prevalence and extrapolation of the number of people living with HBV in the general population of China according to age in 2018

Based on the age structure of national population provided by the National Bureau of Statistics and sub-meta-analysis of HBV infection according to age groups, an estimated 83,864,139 individuals (95% CI: 60,406,793 -110,751,614) were found to be living with HBsAg in China in 2018 [43] (Fig. 4).

## Discussion

Hepatitis B is a major global public health problem particularly in developing countries. The last Chinese National Hepatitis Seroepidemiological Survey was conducted in 2006. Over a decade has passed since 2006, yet no further investigation has been conducted. To obtain the latest national data on hepatitis B in the general population for the assessment of hepatitis B prevalence in China, and to promote effective policy making, a systematic review and meta-analysis was performed covering the years 2013–2017. Having reviewed journal articles in both Chinese and English, this meta-analysis provided a comprehensive and systematic study of the current hepatitis B infection prevalence in China, covering not only the more developed eastern regions, but also those underdeveloped regions such as Tibet. According to our systematic review, the prevalence of HBV infection in the general population in China from 2013 to 2017 was 6.89% (95% *CI*:5.84–7.95%) which is considered as a higher intermediate prevalence. The prevalence rate decreased by 4.04% compared with 2007 and the downward trend was not obvious after 10 years. In 2015, Schweitzer A [5] systematically reviewed the status of chronic HBV infection around the world from 1965 to 2013. In 2017, Zhang WL [44] demonstrated the HBsAg positive rate among the general population in China from 2007 to 2016. Their research indicated that China was still a higher intermediate prevalence area and therefore verified the results of our study. In 2018, an estimated population of approximately 84 million living with HBsAg which has led to many public health problems such as an increase in patients diagnosed with liver cirrhosis and HCC, as it has been proven that patients with HBV infection have a greater risk of HCC [45]. Such a large number of infections also suggested that we should promote policy studies on hepatitis B antiviral treatment, to address the increase in chronic hepatitis B, cirrhosis and HCC patients.

According to our systematic review, in adults older than 20 years, the prevalence of HBV infection was approximately 7% which was higher than that in children. In 2018, it was estimated that more than 90% of the HBV infection population included adults older than 20 years, suggesting that antiviral therapy should be widely adopted to improve the quality of life and the survival rate of infected adults. The high prevalence of HBV infection in individuals aged 20–39 years also suggested that we should continue blocking hepatitis B mother-to-infant transmission as well as plan involving a timely birth dose of hepatitis B vaccine within 24 h considering the high prevalence in women of child-bearing age. The prevalence of HBV infection in males was higher than that in females (5.88% vs 5.05%). This difference might be due to an increased exposure to risk factors, such as occupational risk factors, drug usage, or male homosexuality. This finding also suggested that we should consider the vaccination of hepatitis B vaccine in high risk adults. Compared with the Eastern (6.61% and Central (5.23%) regions, the prevalence of HBV infection was the highest in Western China (8.92%), which was considered a high prevalence area. This conclusion is consistent with the epidemiological survey in 2007 [11]. This difference might be due to different immunization coverages involving timely birth dose and three doses of hepatitis B vaccine in different areas. The immunization coverage of timely birth dose varied widely from 94% in Beijing to only 25% in Tibet, and the three doses of hepatitis B vaccine varied from 100% in Beijing to only 79% in Tibet [46]. These results also showed that plans involving timely birth dose of hepatitis B vaccine within 24 h and immunization coverage of three doses of hepatitis B vaccine should be strictly implemented. The prevalence of HBV infection in rural areas was higher than that in urban areas (5.86% vs 3.29%). This difference might be due to a higher proportion of paid blood donations, and a lower popularity rate of HBV mother-to-infant transmission blocking in rural areas. The higher prevalence of HBV infection in rural and Western areas suggested that more policy and finance support should be given to promote disease prevention and treatment in those areas, and various control measures should be implemented in different provinces across China.

There are three main limitations of this review. First, studies were conducted in various geographical areas, targeting different populations, so the results of heterogeneity test indicated that the studies were significantly heterogeneous. However, subgroup analysis was used to minimize this heterogeneity and provided more details on HBV infection. Second, it should be noted that only four studies reported the prevalence of HBV infection in the general population in 31 provinces at a national level. At a regional level, little is known about HBV infection in the general population in all provinces in China. Third, because not all the full-text articles included sex, age, region and other information required for subgroup analysis, some of the articles with higher positive rates were not included in subgroup analysis. Despite resulting in a higher pooled prevalence of HBV infection rate and a lower rate in subgroup analysis, the above situation also provided more details on HBV infection in China.

## Conclusion

China was classified as a higher intermediate prevalence area (5–7.99%), of which more than 90% of HBV infection population included adults older than 20 years. These results provided two valuable pieces of information. First, the high prevalence of hepatitis B in women of child-bearing age indicated that blocking hepatitis B mother-to-infant transmission and the immunization plan involving a timely birth dose of hepatitis B vaccine within 24 h should be strictly implemented. Second, although the number of HBV-infected populations has decreased over the past 10 years, an estimated population of 84 million were still infected which is more than the number of people in any country of Europe. Improving the quality of life and survival rate of the infected population through antiviral therapy and high risk adult vaccination will be the priority of our future work. More policy and finance support should be provided for prevention and treatment in rural and Western areas, and various control measures should be implemented in different provinces across China.

## Additional files

Additional file 1: **Table S1.** Cross-Sectional/Prevalence Study Quality Assessment Forms (AHRQ). (DOCX 16 kb)

Additional file 2: **Table S2.** Quality assessment of eligible studies. (DOCX 18 kb)

## Data Availability

Not applicable.

## Abbreviations

AHRQ: Agency for Healthcare Research and Quality
CBM: Chinese Biomedical Database
CDC: Centers for Disease Control and Prevention
CNKI: China National Knowledge Infrastructure
HBsAg: Hepatitis B surface antigen
HBV: Hepatitis B virus
HCC: Hepatic Cirrhosis and Hepatocellular Carcinoma
PRISMA: Preferred Reporting Items for Systematic Reviews and Meta-Analyses
