# Supplementary material for: Hepatitis B infection in the general population of China: a systematic review and meta-analysis
Source: BMC Infect Dis. 2019 Sep 18;19:811. doi: 10.1186/s12879-019-4428-y (PMC6751646; doi:10.1186/s12879-019-4428-y)
Supplement: Supplementary file 2 — Table S2. Quality assessment of eligible studies. (DOCX 18 kb) [file 12879_2019_4428_MOESM2_ESM.docx]

**ADDITIONAL FILE 2**

**Table S2 Quality assessment of eligible studies.**

| **Author, Year** | **Q1** | **Q2** | **Q3** | **Q4** | **Q5** | **Q6** | **Q7** | **Q8** | **Q9** | **Q10** | **Score** |
| --- | --- | --- | --- | --- | --- | --- | --- | --- | --- | --- | --- |
| Min DY, 2016[16] | Y | UC | Y | Y | UC | N | N | N | N | Y | 4 |
| Xia W, 2015[17] | Y | UC | Y | Y | UC | N | N | N | N | Y | 4 |
| Cai HL, 2017[18] | Y | Y | Y | Y | UC | N | N | N | N | N | 4 |
| Bai S, 2017[19] | Y | N | Y | Y | UC | N | N | N | N | Y | 4 |
| Wang FZ, 2017[20] | Y | UC | Y | Y | UC | Y | N | N | N | Y | 5 |
| Chen YH, 2017[21] | Y | UC | Y | Y | UC | Y | N | N | N | Y | 5 |
| Gao P, 2016[22] | Y | UC | Y | Y | UC | Y | N | Y | N | Y | 6 |
| Su FY, 2015[23] | Y | UC | Y | Y | UC | N | N | N | N | Y | 4 |
| Yang BF, 2013[24] | Y | Y | Y | Y | UC | Y | N | Y | N | Y | 7 |
| Liu J, 2014[25] | Y | UC | Y | Y | UC | Y | N | N | N | Y | 5 |
| Ren H, 2013[26] | Y | UC | Y | Y | UC | N | N | N | N | Y | 4 |
| Cheng JQ, 2013[27] | Y | UC | Y | Y | UC | N | N | N | N | Y | 4 |
| He HY, 2014[28] | Y | UC | Y | Y | UC | Y | N | N | N | Y | 5 |
| Guo YH, 2017[29] | Y | Y | Y | Y | UC | UC | N | Y | N | Y | 6 |
| Liu J, 2017[30] | Y | UC | Y | Y | UC | UC | N | Y | N | Y | 5 |
| Liu JY, 2017[31] | Y | UC | Y | Y | UC | Y | N | Y | N | Y | 6 |
| Yang SG,2017[32] | Y | UC | Y | Y | UC | UC | Y | Y | N | Y | 6 |
| Chen P, 2017[33] | Y | Y | Y | Y | UC | Y | Y | Y | N | Y | 8 |
| Zeng FF, 2016[34] | Y | Y | Y | Y | UC | Y | N | Y | N | Y | 7 |
| Xin XN, 2016[35] | Y | Y | Y | Y | UC | UC | N | N | N | Y | 5 |
| Liu J, 2016[36] | Y | UC | Y | N | UC | Y | N | N | N | Y | 4 |
| Zhang Q, 2016[37] | Y | Y | Y | Y | UC | Y | N | N | N | Y | 6 |
| Guo YH, 2015[38] | Y | UC | Y | Y | UC | Y | N | N | N | Y | 5 |
| Huang P, 2015[39] | Y | UC | Y | Y | UC | Y | N | N | N | Y | 5 |
| Ji ZH, 2014 [40] | Y | UC | Y | Y | UC | Y | N | N | N | Y | 5 |
| Liao XY, 2014[41] | Y | N | N | Y | UC | Y | N | N | N | Y | 4 |
| Zhang Y, 2013[42] | Y | UC | Y | Y | UC | N | N | N | N | Y | 4 |

Y: Yes; N: No; UC: Unclear.
